# Supplementary material for: The Chimeric Adenovirus (Ad5/35) Expressing Engineered Spike Protein Confers Immunity against SARS-CoV-2 in Mice and Non-Human Primates
Source: Vaccines (Basel). 2022 Apr 30;10(5):712. doi: 10.3390/vaccines10050712 (PMC9147121; doi:10.3390/vaccines10050712)
Supplement: Supplementary file 1 [file vaccines-10-00712-s001.zip › vaccines-1693019-supplementary.pdf]

Supplementary Figure S1. Construction of candidate Adenovirus vectored vaccine against SARS-CoV-2

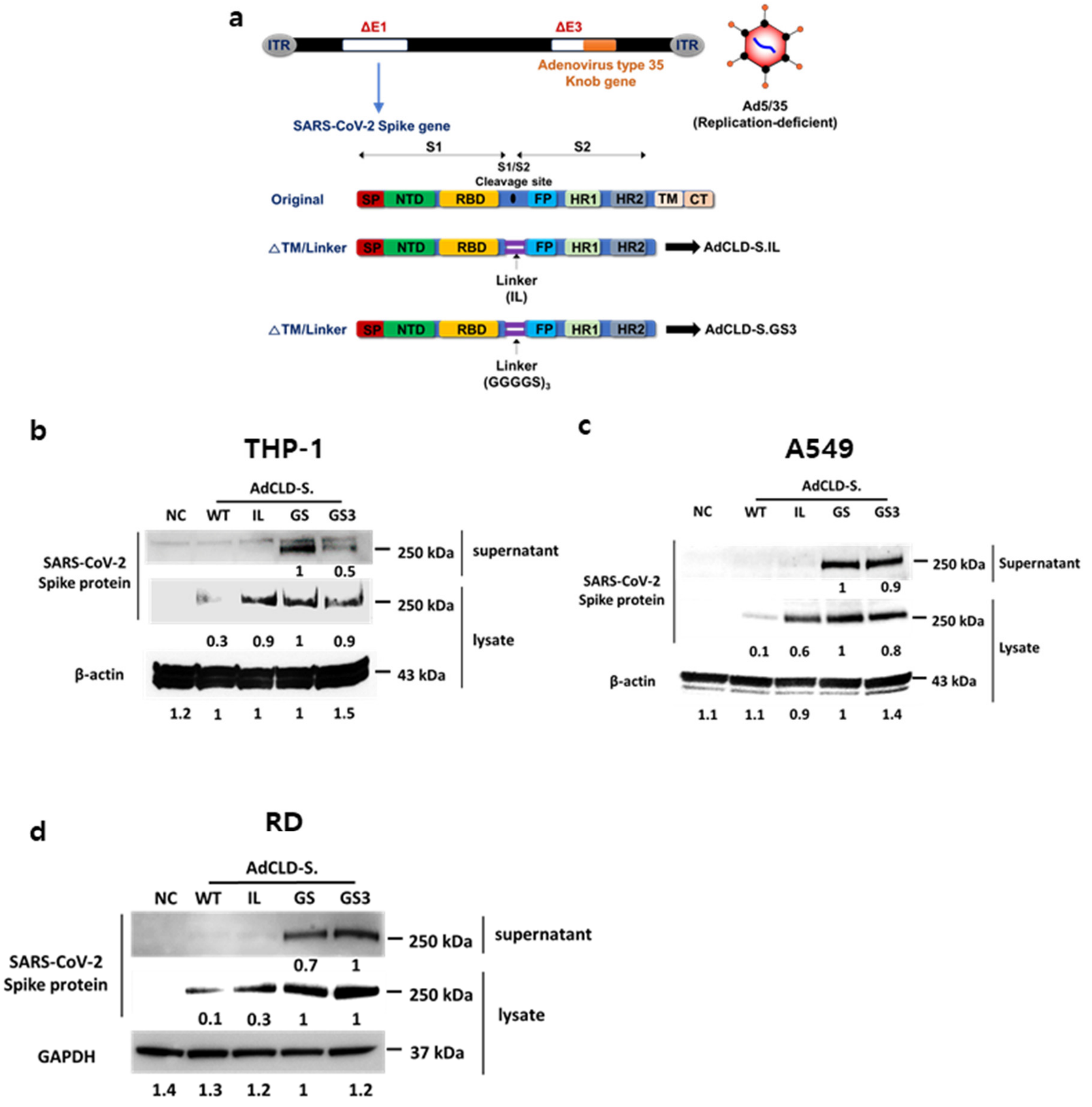

(**a**) Schematic diagram of the structure of adenovirus vectored vaccine harbored with modified SARS-CoV-2 S protein. (**b–d**) AdCLD-S.WT, AdCLD-S.IL, AdCLD-CoV19 or AdCLD-S.GS3 was transformed into THP-1 (**b**), A549 (**c**), RD (**d**) cell line (each $2.5x10^5$ cells) at each 25 MOI. The accumulation level of S protein secretion was verified by Western blot analysis after harvesting supernatant (40 ug/well) and cell lysate (40 ug/well) of cells transformed with each vaccine candidates. SP, signal sequence; NTD, N-terminal domain RBD, receptor-binding domain; FP, fusion peptide; HR1, heptad repeat 1; HR2, heptad repeat 2; TM, transmembrane domain; CT, cytoplasmic tail; NC, Negative control

Supplmentary Figure S2. Establishment of hACE2-expressing cell line for neutralizing antibody assay

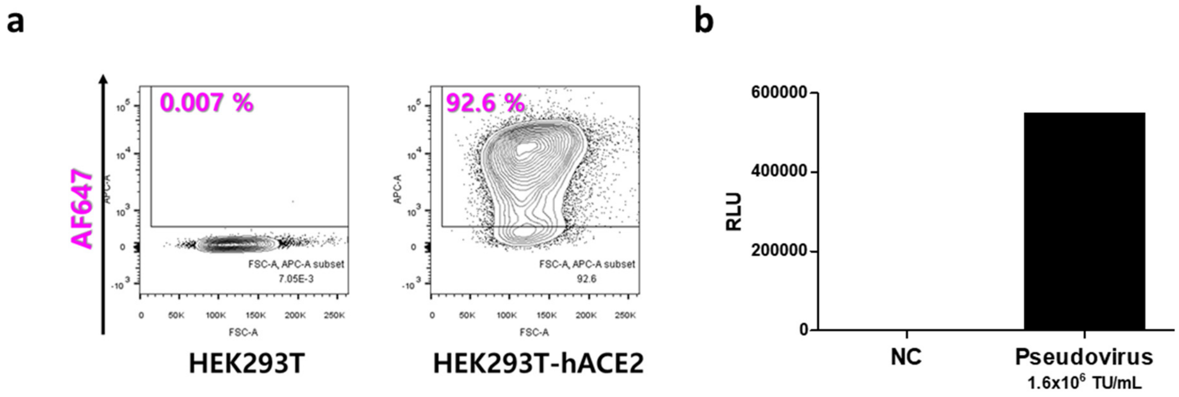

The HEK293T-hACE2 cell line was established by transducing a lentivirus harboring a human ACE2 gene into HEK293T cells. (**a**) HEK293T-hACE2 cell line was reacted with AF647 conjugated anti-hACE2 antibody and analyzed by flow cytometry. (**b**) The neutralizing antibody assay using HEK293T-hACE2 was verified through infection of the pseudotyped lentivirus (Wuhan-Hu-1 wild-type, $1.6x10^{6}$ TU/mL) loaded with a reporter gene, luciferase. NC, Negative Control; RLU, Relative Light Unit.

Supplementary Figure S3. Immunogenicity of AdCLD-CoV19 in macaques before SARS-CoV-2 challenge

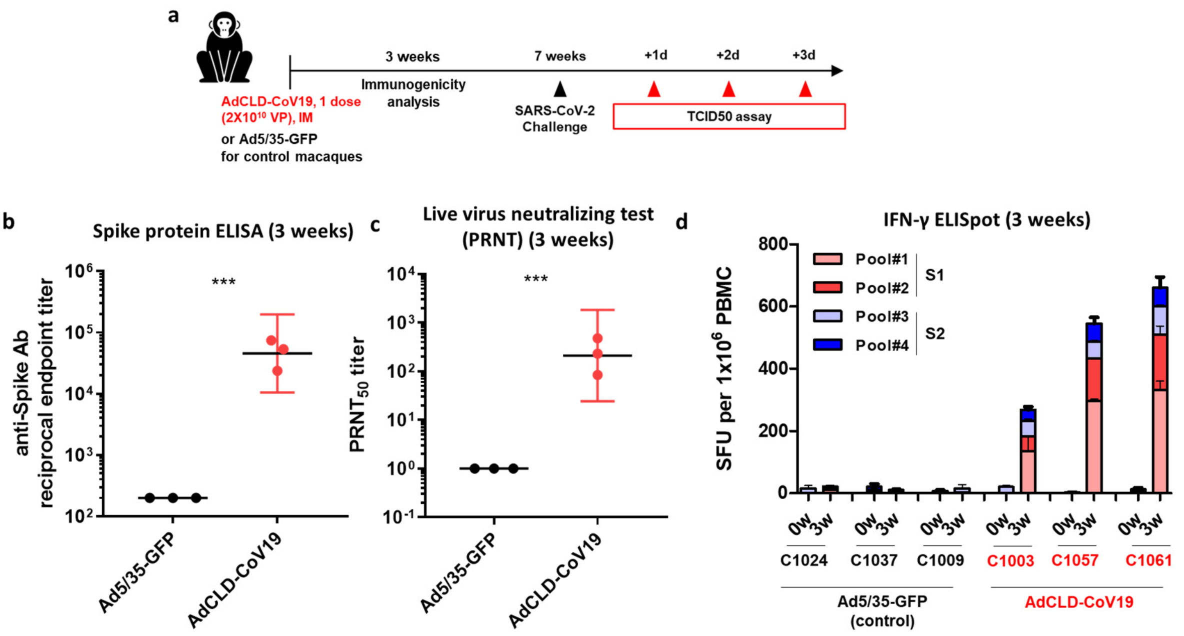

(**a**) AdCLD-CoV19-vaccinated cynomolgus macaques and control vector (Ad5/35-GFP)-administered macaques at day 0. 3 weeks after vaccination, serums and PBMCs were collected from macaques and immunogenicity analysis were conducted as follows: (**b**) binding Ab titers by ELISA, (**c**) Neutralizing Ab titers by Plaque-reduction neutralizing test (PRNT), and (**d**) cellular immunity by ELISpot. ***, $p < 0.001$; ns, not significant.

Supplementary Figure S4. The comparison of the properties of AdCLD-CoV19 and AdCLD-CoV19-1

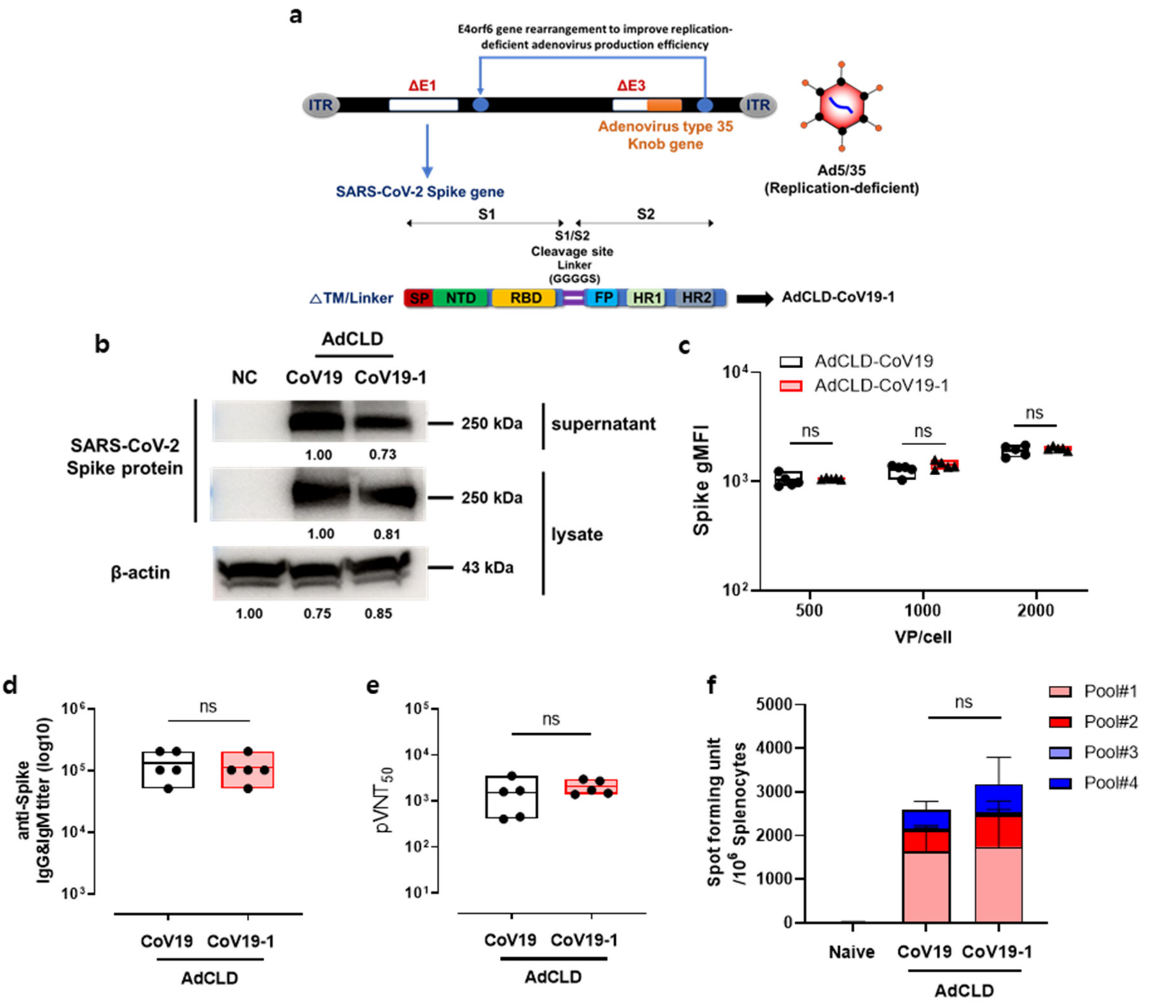

(**a**) AdCLD-CoV19-1 structural schematic diagram. The antigen in AdCLD-CoV19-1 is structurally identical to AdCLD-CoV19, and the E4orf6 gene was rearranged to the E1 deletion region to enhance replication-deficient adenovirus production. (**b**) At each 25 MOI, AdCLD-CoV19 or AdCLD-CoV19-1 was transformed into A549 cell line ($2.5x10^5$ cells). Western blot analysis verified the level of S protein secretion accumulation after harvesting supernatant (40 ug/well) and cell lysate (40 ug/well) from transformed cells.(**c**)AdCLD-CoV19 and AdCLD-CoV19-1 were infected at 500, 1000, and 2000 VP/cell in the A549 cell line. The antigen expression levels of each vaccine were compared by flow cytometry (geometric mean; gMFI). (**d–f**) The 8-week sera of BALB/c mice immunized with each 1x109 VP AdCLD-CoV19 or AdCLD-CoV19-1 were analyzed for differences between vaccines by ELISA (**d**) and neutralizing antibody activity assay (**e**). (**f**) IFN-γ-secreting T cells were measured by ELISpot assay from vaccinated Balb/c mice at 4 weeks splenocytes that stimulated with each peptide pools. All graphs,

except the (**f**) graph, are displayed with floating bar (min to max) boxes and mean values indicated by inner lines. ns, not significant. SP, signal sequence; NTD, N-terminal domain RBD, receptor-binding domain; FP, fusion peptide; HR1, heptad repeat 1; HR2, heptad repeat 2; TM, transmembrane domain; CT, cytoplasmic tail; NC, Negative control. ns, not significant.

Supplementary Figure S5. Western blotting raw data

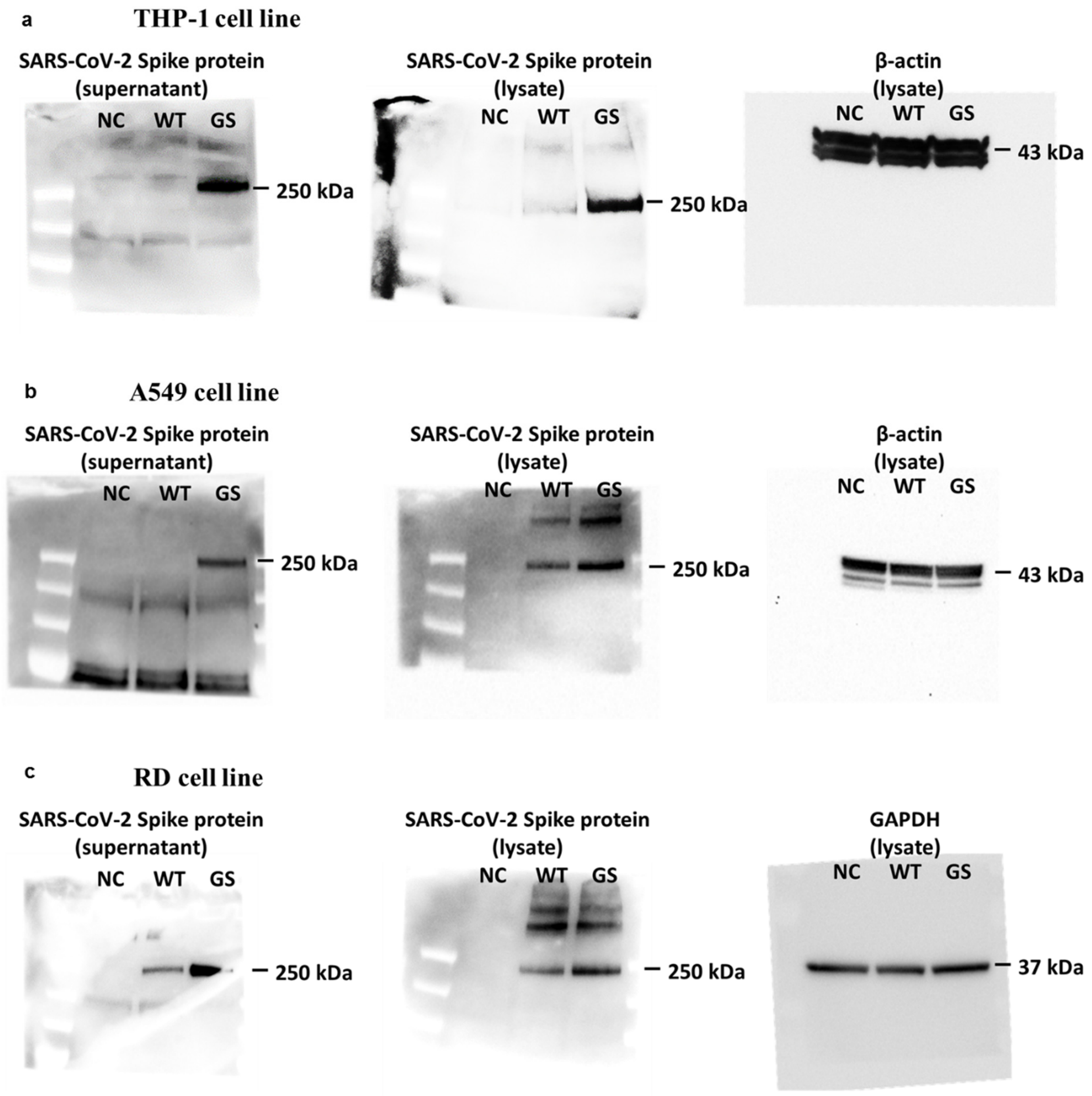

d

THP-1 cell line

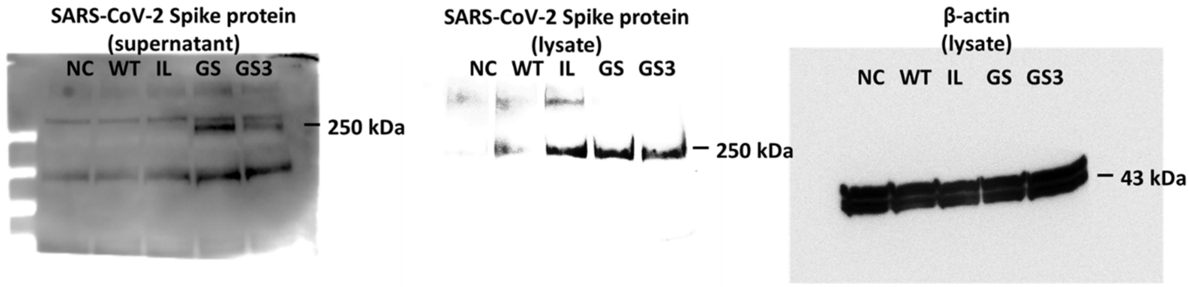

e

A549 cell line

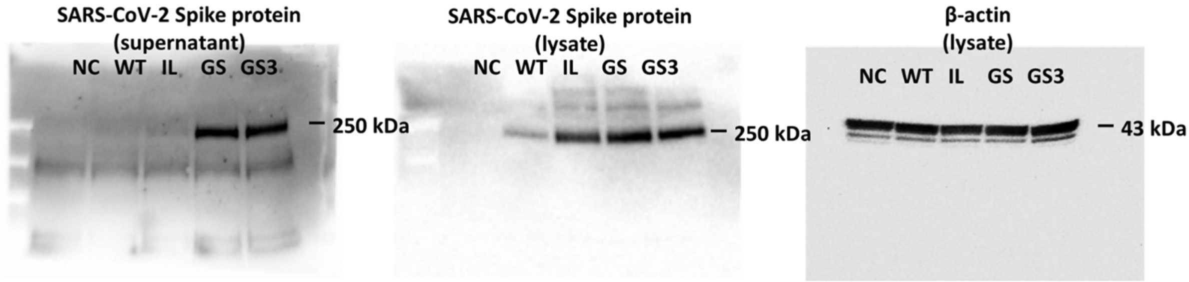

f

RD cell line

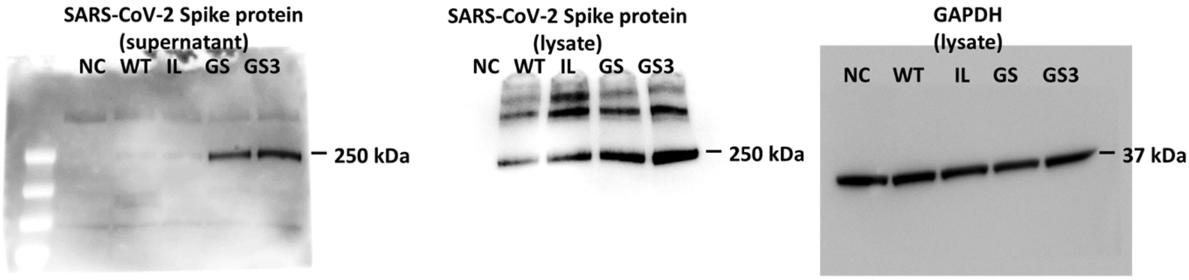

g

A549 cell line

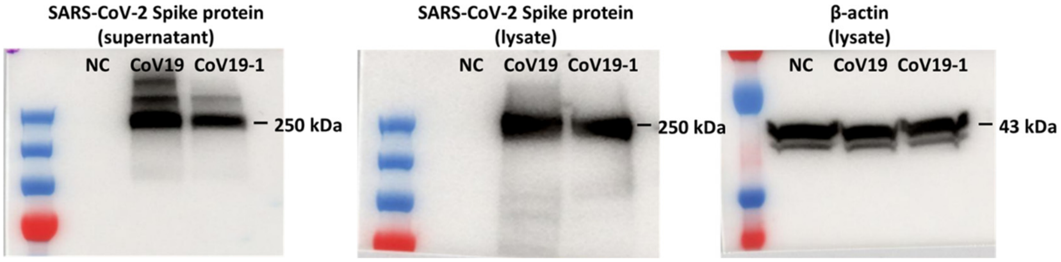

Uncropped images of western blots shown in Figure 1b–d **(a–c)**, Supplementary Figure S1b–d **(d–f)** and Supplementary Figure S4b (**g**).

Supplementary Table S1. Statistical summary of humoral immunity induced by AdCLD-CoV19 in mice. Related to Figure 2

[Figure 2a and 2c] Spike protein ELISA (GMT with 95% CI)

Vaccination	Weeks post vaccination						
	0w	2w	3w	4w	5w	6w	7w
**AdCLD-S.WT (1×10^9 VP) (N=5)**	200 (200, 200)	33779 (21083, 54123)	29407 (14313, 60419)	44572 (21694, 91578)	33779 (12661, 90121)	51200 (27859, 94096)	33779 (12661, 90121)
**AdCLD-CoV19 (1×10^9 VP) (N=5)**	200 (200, 200)	44572 (21694, 91578)	58813 (40024, 86424)	117627 (57251, 241675)	135118 (84331, 216490)	135118 (84331, 216490)	155209 (96870, 248682)
**AdCLD-CoV19 (2×10^8 VP) (N=5)**	200 (200, 200)	22286 (10847, 45789)	44572 (21694, 91578)	58813 (28625, 120837)	58813 (22910, 150983)	102400 (43303, 242146)	102400 (55718, 188192)

[Figure 2b] Pseudovirus neutralization test (GMT with 95% CI)

Vaccination	Weeks post vaccination			
	0w	3w	5w	7w
**AdCLD-S.WT (1×10^9 VP) (N=4)**	28.75 (11.26, 73.39)	62.47 (23.31, 167.4)	142.4 (45.39, 446.8)	226.2 (61.96, 825.6)
**AdCLD-CoV19 (1×10^9 VP) (N=4)**	37.23 (18.06, 76.74)	179.7 (73.71, 438.2)	516.8 (383.6, 696.4)	1468 (1104, 1951)

[Figure 2d] Pseudovirus neutralization test (GMT with 95% CI)

Vaccination	Weeks post vaccination			
	0w	2w	4w	6w
**AdCLD-CoV19 (1×10^9 VP) (N=4)**	33.97 (17.88, 64.55)	68.85 (30.19, 157.1)	403.0 (339.1, 479.1)	812.4 (201.7, 3273)
**AdCLD-CoV19 (2×10^8 VP) (N=4)**	38.48 (19.55, 75.72)	74.67 (31.75, 175.6)	252.8 (145.8, 438.2)	411.3 (211.8, 798.7)

Supplementary Table S2. Statistical summary of humoral immunity induced by AdCLD-CoV19 in non-human primates. Related to Figure 3

[Figure 3a and 3d] Spike protein ELISA (GMT* with min to max)

Vaccination	Weeks post vaccination				
	**0w**	**2w**	**3w**	**4w**	**5w**
**AdCLD-S.WT (1×10^{11} VP) (N=2)**	100 (100, 100)	36204 (25600, 51200)	25600 (25600, 25600)	25600 (25600, 25600)	25600 (25600, 25600)
**AdCLD-CoV19 (1×10^{11} VP) (N=2)**	100 (100, 100)	72408 (51200, 102400)	51200 (51200, 51200)	51200 (51200, 51200)	51200 (25600, 102400)
**AdCLD-CoV19 (2×10^{10} VP) (N=3)**	100 (100, 100)	64508 (51200, 102400)	64508 (51200, 102400)	25600 (12800, 51200)	32254 (12800, 51200)
**AdCLD-CoV19 (4×10^{9} VP) (N=3)**	100 (100, 100)	10159 (6400, 12800)	16127 (12800, 25600)	12800 (12800, 12800)	12800 (12800, 12800)

[Figure 3b] Pseudovirus neutralization test (GMT with min to max)

Vaccination	Weeks post vaccination				
	**0w**	**2w**	**3w**	**4w**	**5w**
**AdCLD-S.WT (1×10^{11} VP) (N=2)**	18.99 (17.85, 20.20)	215.2 (127.6, 363.1)	185.3 (110.2, 311.7)	171.2 (103.1, 284.3)	100.8 (92.55, 109.8)
**AdCLD-CoV19 (1×10^{11} VP) (N=2)**	14.62 (10.00, 21.38)	378.1 (255.4, 559.7)	297.1 (224.9, 392.4)	415.2 (358.3, 481.1)	352.9 (314.0, 396.6)

[Figure 3c] Focus reduction neutralization test (individual data)

Vaccination	Weeks post vaccination
	**3w**
**AdCLD-S.WT (1×10^{11} VP) (N=2)**	Monkey 1: 1104<br>Monkey 2: 561.1
**AdCLD-CoV19 (1×10^{11} VP) (N=2)**	Monkey 3: 1372<br>Monkey 4: 2701

[Figure 3e] Pseudovirus neutralization test (GMT with min to max)

Vaccination	Weeks post vaccination				
	0w	2w	3w	4w	5w
**AdCLD-CoV19 (1×10^{11} VP) (N=2)**	32.72 (28.19, 37.97)	449.9 (235.4, 859.9)	353.6 (217.2, 575.6)	415.2 (358.3, 481.1)	352.9 (314.0, 396.6)
**AdCLD-CoV19 (2×10^{10} VP) (N=3)**	46.85 (39.85, 56.85)	219.4 (115.1, 306.5)	131.7 (85.58, 230.3)	229.5 (115.4, 611.4)	265.9 (188.7, 453.5)
**AdCLD-CoV19 (4×10^{9} VP) (N=3)**	48.50 (46.28, 51.53)	45.23 (32.51, 65.91)	47.23 (31.55, 77.90)	37.14 (26.58, 63.11)	57.14 (41.65, 79.02)

* 95% CI was not considered in NHP studies due to a small number of animal.

Supplementary Table S3. Materials and Methods primer list

Candidates	Orientation	Sequence (5' → 3')	Mer
AdCLD-S.WT	Forward	TCC AGC CTC CGA TTT GCC ACC ATG TTT GTG TTC CTG G	37
	Reverse	GAT TAT GAT CAA TTT TCA TGG CCA CTT GAT GTA TTG TTC	39
TNSPRRAR → TILR (AdCLD-S.IL)	Forward	AGA CCA TCC TCA GGT CTG TGG CAA GCC AGA G	31
	Reverse	CCT CCT ACC AGA CCC AGA CCA TCC TCA GGT	30
TNSPRRAR → TGGGGSR (AdCLD-S.GS or AdCLD-CoV19)	Forward	CGG TGG CGG TGG GTC GAG GTC TGT GGC AAG CCA GAG	36
	Reverse	GAC CCA CCG CCA CCG GTC TGG GTC TGG TAG GAG G	34
TNSPRRAR →T(GGGGS)3R (AdCLD-S.GS3)	Forward	GGG CGG TGG TGG GTC GGG TGG CGG CGG TTC CAG GTC TGT GGC AAG CCA GAG	51
	Reverse	GAC CCA CCA CCG CCC GAC CCA CCG CCA CCG GTC TGG GTC TGG TAG GAG G	49

Supplementary Table S4. Sequences of SARS-CoV-2 spike protein peptide library

Peptide ID	Sequence (N' - C')	Subunit	Pool (No. of peptides in pool)
1	MFVFLVLLPLVSSQC	S1	Pool #1 (49)
2	LPLVSSQCVNLTTRT		
3	CVNLTT RTQLPPAYT		
4	TQLPPAYTN SFTRGV		
5	TNSFTRGVYYPDKVF		
6	VYYPDKVFRSSVLHS		
7	FRSSVLHSTQDLFLP		
8	STQDLFLPFFSNVTW		
9	PFFSNVTWFHAIHVS		
10	WFHAIHVSGTNGTKR		
11	SGTNGTKRFDNPVLP		
12	RFDNPVLPFNDGVYF		
13	PFNDGVYFASTEKSN		
14	FASTEKSNIIRGWIF		
15	NIIRGWIFGTTLDSK		
16	FGTTLDSKTQSLLIV		
17	KTQSLLIVNNATNVV		
18	VNNATNVVIKVCEFQ		
19	VIKVCEFQFCNDPFL		
20	QFCNDPFLGVYYHKN		
21	LGVYYHKNNKSWMES		
22	NNKSWMESEFRVYSS		
23	SEFRVYSSANNCTFE		
24	SANNCTFEYVSQPFL		
25	EYVSQPFLMDLEGKQ		
26	LMDLEGKQGNFKNLR		
27	QGNFKNLREFVFKNI		
28	REFVFKNIDGYFKIY		
29	IDGYFKIYSKHTPIN		
30	YSKHTPINLVRDLPQ		
31	NLVRDLPQGFSALEP		
32	QGFSALEPLVDLPIG		
33	PLVDLPIGINITRFQ		
34	GINITRFQTLLALHR		
35	QTLLALHRSYLTPGD		

36	RSYLTPGDSSSGWTA		
37	DSSSGWTAGAAAYYV		
38	AGAAAYYVGYLQPRT		
39	VGYLQPRTFLLKYNE		
40	TFLLKYNENGTITDA		
41	ENGTITDAVDCALDP		
42	AVDCALDPLSETKCT		
43	PLSETKCTLKSFTVE		
44	TLKSFTVEKGIYQTS		
45	EKGIYQTSNFRVQPT		
46	SNFRVQPTESIVRFP		
47	TESIVRFPNITNLCP		
48	PNITNLCPFGEVFNA		
49	PFGEVFNATRFASVY		
50	ATRFASVYAWNRKRI		**Pool #2 (48)**
51	YAWNRKRISNCVADY		
52	ISNCVADYSVLYNSA		
53	YSVLYNSASFSTFKC		
54	ASFSTFKCYGVSPTK		
55	CYGVSPTKLNDLCFT		
56	KLNDLCFTNVYADSF		
57	TNVYADSFVIRGDEV		
58	FVIRGDEVRQIAPGQ		
59	VRQIAPGQTGKIADY		
60	QTGKIADYNYKLPDD		
61	YNYKLPDDFTGCVIA		
62	DFTGCVIAWNSNNLD		
63	AWNSNNLDSKVGGNY		
64	DSKVGGNYNYLYRLF		
65	YNYLYRLFRKSNLKP		
66	FRKSNLKPFERDIST		
67	PFERDISTEIYQAGS		
68	TEIYQAGSTPCNGVE		
69	STPCNGVEGFNCYFP		
70	EGFNCYFPLQSYGFQ		
71	PLQSYGFQPTNGVGY		
72	QPTNGVGYQPYRVVV		
73	YQPYRVVVLSFELLH		
74	VLSFELLHAPATVCG		
75	HAPATVCGPKKSTNL		

76	GPKKSTNLVKNKCVN		
77	LVKNKCVNFNFNGLT		
78	NFNFNGLTGTGVLTE		
79	TGTGVLTESNKKFLP		
80	ESNKKFLPFQQFGRD		
81	PFQQFGRDIADTTDA		
82	DIADTTDAVRDPQTL		
83	AVRDPQTLEILDITP		
84	LEILDITPCSFGGVS		
85	PCSFGGVSVITPGTN		
86	SVITPGTNTSNQVAV		
87	NTSNQVAVLYQDVNC		
88	VLYQDVNCTEVPVAI		
89	CTEVPVAIHADQLTP		
90	IHADQLTPTWRVYST		
91	PTWRVYSTGSNVFQT		
92	TGSNVFQTRAGCLIG		
93	TRAGCLIGAEHVNNS		
94	GAEHVNNSYECDIPI		
95	SYECDIPIGAGICAS		
96	IGAGICASYQTQTNS		
97	SYQTQTNSPRRARSV		

Peptide ID	Sequence (N' - C')	Subunit	Pool (No. of peptides in pool)
98	SPRRARSVASQSIIA	S2	Pool #3 (38)
99	VASQSIIAYTMSLGA		
100	AYTMSLGAENSVAYS		
101	AENSVAYSNNSIAIP		
102	SNNSIAIPTNFTISV		
103	PTNFTISVTTEILPV		
104	VTTEILPVSMTKTSV		
105	VSMTKTSVDCTMYIC		
106	VDCTMYICGDSTECS		
107	CGDSTECSNLLLQYG		
108	SNLLLQYGSFCTQLN		
109	GSFCTQLNRALTGIA		
110	NRALTGIAVEQDKNT		
111	AVEQDKNTQEVFAQV		
112	TQEVFAQVKQIYKTP		
113	VKQIYKTPPIKDFGG		
114	PPIKDFGGFNFSQIL		
115	GFNFSQILPDPSKPS		
116	LPDPSKPSKRSFIED		
117	SKRSFIEDLLFNKVT		
118	DLLFNKVTLADAGFI		
119	TLADAGFIKQYGDCL		
120	IKQYGDCLGDIAARD		
121	LGDIAARDLICAQKF		
122	DLICAQKFNGLTVLP		
123	FNGLTVLPPLLTDEM		
124	PPLLTDEMIAQYTSA		
125	MIAQYTSALLAGTIT		
126	ALLAGTITSGWTFGA		
127	TSGWTFGAGAALQIP		
128	AGAALQIPFAMQMAY		
129	PFAMQMAYRFNGIGV		
130	YRFNGIGVTQNVLYE		
131	VTQNVLYENQKLIAN		
132	ENQKLIANQFNSAIG		
133	NQFNSAIGKIQDSLS		
134	GKIQDSLSSTASALG		

135	SSTASALGKLQDVVN		
136	GKLQDVVNQNAQALN		Pool #4 (38)
137	NQNAQALNTLVKQLS		
138	NTLVKQLSSNFGAIS		
139	SSNFGAISSVLNDIL		
140	SSVLNDILSRLDKVE		
141	LSRLDKVEAEVQIDR		
142	EAEVQIDRLITGRLQ		
143	RLITGRLQSLQTYVT		
144	QSLQTYVTQQLIRAA		
145	TQQLIRAAEIRASAN		
146	AEIRASANLAATKMS		
147	NLAATKMSECVLGQS		
148	SECVLGQSKRVDFCG		
149	SKRVDFCGKGYHLMS		
150	GKGYHLMSFPQSAPH		
151	SFPQSAPHGVVFLHV		
152	HGVVFLHVTYVPAQE		
153	VTYVPAQEKNFTTAP		
154	EKNFTTAPAICHDGK		
155	PAICHDGKAHFPREG		
156	KAHFPREGVFVSNGT		
157	GVFVSNGTHWFVTQR		
158	THWFVTQRNFYEPQI		
159	RNFYEPQIITTDNTF		
160	IITTDNTFVSGNCDV		
161	FVSGNCDVVIGIVNN		
162	VVIGIVNNTVYDPLQ		
163	NTVYDPLQPELDSFK		
164	QPELDSFKEELDKYF		
165	KEELDKYFKNHTSPD		
166	FKNHTSPDVDLGDIS		
167	DVDLGDISGINASVV		
168	SGINASVVNIQKEID		
169	VNIQKEIDRLNEVAK		
170	DRLNEVAKNLNESLI		
171	KNLNESLIDLQELGK		
172	IDLQELGKYEQYIKW		
173	KYEQYIKWPWYIWLG		
174	WPWYIWLGFIAGLIA	**TM&CT**	

175	GFIAGLIAIVMVTIM		**Not used in this study because the vaccine candidates do not contain the transmembrane-cytoplasmic tail of spike protein**
177	MLCCMTSCCSCLKGC		
178	CCSCLKGCCSCGSCC		
179	CCSCGSCCKFDEDDS		
180	CKFDEDDSEPVLKGV		
181	SEPVLKGVKLHYT		
